# Standardization of experimental model regarding star fruit intoxication in Wistar rats suffering with nephropathy

**DOI:** 10.1590/ACB360204

**Published:** 2021-02-22

**Authors:** Layla Alves Rodrigues da Silva, Renato Figueiredo Santana, Eduardo Achar, Gerson Ballester, Sandra Regina Mota Ortiz, Marcelo Augusto Fontenelle Ribeiro

**Affiliations:** 1 Fellow Master Degree. Instituto de Assistência Médica ao Servidor Público Estadual – Postgraduate Program in Health Science – São Paulo (SP), Brazil.; 2 PhD. Universidade Cidade de São Paulo and Universidade São Judas Tadeu – São Paulo (SP), Brazil.; 3 PhD. Universidade Cidade de São Paulo and Universidade de São Caetano do Sul – São Paulo (SP), Brazil.; 4 PhD. Universidade Cidade de São Paulo – São Paulo (SP), Brazil.; 5 PhD Professor. Universidade São Judas Tadeu and Professor – Postgraduate Program in Aging Science; Instituto de Assistência Médica ao Servidor Público Estadual – Postgraduate Program in Health Science – São Paulo (SP), Brazil.; 6 Full Professor. Pontifícia Universidade Católica de São Paulo – Department of Surgery – Sorocaba (São Paulo). Professor. Instituto de Assistência Médica ao Servidor Público Estadual – Postgraduate Program in Health Science – São Paulo (SP), Brazil.

**Keywords:** Averrhoa, Seizures, Kidney Diseases, Electroencephalography, Rats

## Abstract

**Purpose:**

To present a model to reproduce the clinical condition, in order to better understand the pathophysiology of neurological impairment related to intoxication.

**Methods:**

Twenty-five Wistar rats were used and divided into five groups: Shaw group (WHI), water gavage group (WGV), star fruit gavage group (SGV), nephropathic group with water gavage (NPW), nephropathic group with star fruit gavage (NPS).Nephropathic groups were submitted to surgery, developing nephropathy. After surgery, they received preestablished gavage with star fruit juice or water. The electroencephalographic records were evaluated in the experimental nephropathic group that received gavage of star fruit juice.

**Results:**

To assess the induction of neurotoxicity using electroencephalographic data, the NPS group demonstrated the presence of epileptic seizures associated with star fruit intoxication.

**Conclusions:**

The experimental model herein presented was adequate to reproduce the clinical condition experienced by nephropathic patients who ingest star fruit juice, establishing, thus, an experimental model utterly important for the study of the neurological toxicity process.

## Introduction

Star fruit (*Averrhoa carambola*) is a fruit cultivated in tropical countries, such as Brazil^1^^,^ 2. The first case report describing an association between the fruit ingestion and the intoxication process in patients with kidney disorders was done by Martin *et al.* 3. The intoxication can be presented in three different levels: mild, moderate or severe, varying according to the clinical manifestation presented by patients such as hiccups, seizures, coma and even death^4^^,^ 5.

Reports indicate that there are two possible theories related to pathophysiology: the first suggests that caramboxin, a specific star fruit toxin, would be able to inhibit GABAergic receptors and increase neuronal excitability, while the second concerns the role of the oxalic acid (oxalate) in inducing neurotoxicity^6^^,^ 7.

The gamma aminobutyric acid (GABA) is the main pathway with neurotransmission inhibitory activity in the vertebrate central nervous system, where there can be found two different types of receptors: the GABA_A_ ionotropic, which correspond to the permeable chlorine ion channels, and the GABA_B_ metabotropic receptors, that are associated with protein G and regulate the potassium and calcium channels activity^8^^,^ 9. In contrast, the glutamatergic pathway is the main pathway with excitatory activity of the central nervous system and its receptors are divided into two groups. First there are the iGluRs, that are the ionotropic receptors and the second group is the one concerning the mGluRs, which are metabotropic receptors that generate slower postsynaptic responses^10^.

Some genes are used to reveal increased levels of cellular activity, being the nuclear protein c-Fos encoded by one of these genes. The c-Fos was the first proto-oncogene to be activated with regard to the expression modulation associated with epileptic seizures, suggesting neuronal activity^11^^,^ 12.

The instability that the neurotoxin induces in the neurotransmitters found in the glutamatergic and/or GABAergic pathways can result in increased cerebral excitability and, with this, potentially lead to a rise in the c-Fos nuclear protein expression, thus making the implementation of an experimental model substantial to further investigation regarding activated neural sites when it comes to intoxication.

## Methods

All experiments respected the rules and precepts of the Brazilian College of Animal Experimentation (COBEA) in order to avoid and minimize animal suffering as much as possible, with research protocol No. 001/2015 approved by the Animal Use Ethics Committee of Universidade Cidade de São Paulo (CEUA-UNICID) on 08/19/2015.

Twenty-five male and adults Wistar rats (weighing between 250 and 350 g) were used in the experiment. The animals were kept in the UNICID vivarium at the Neuroscience Research Center (NUPEN), housed in polypropylene cages (30 × 40 × 18 cm), in rooms with ventilation system (23 **±** 2 °C) under a 12/12 light-dark cycle, with food and water at ease.

The trial design was carried on by dividing the animals into five groups:

- Shaw group (WHI): animals that have not received any type of experimental procedure;
- Water gavage group (WGV): received a water gavage procedure on experimental days 9 and 10;
- Star fruit gavage group (SGV): received a star fruit gavage procedure on experimental days 9 and 10;
- Nephropathic group with water gavage (NPW): on day 8 the animals underwent bilateral ureteral obstruction surgery (BUO) and received a water gavage procedure on experimental days 9 and 10;
- Nephropathic group with star fruit gavage (NPS): on day 1, electrode implant surgery was performed to record the electroencephalographic pattern.

Experimental day 1 was considered the same for all groups.

On day 8, the animals underwent BUO and received a gavage procedure with star fruit on experimental days 9 and 10. On day 10th, the EEG was recorded in order to validate the visual/behavioral analysis that showed the convulsive crisis present in this group (NPS) and not expressed in the other groups. Arterial blood was collected in all groups (transcardiac) in order to measure urea and plasmatic creatinine on day 10 (Table 1).

**Table 1:** Procedures carried on according to time. Shaw group (WHI); water gavage group (WGV); star fruit gavage group (SGV); nephropathic group with water gavage (NPW); nephropathic group with star fruit gavage (NPS); bilateral ureteral obstruction surgery (BUO).

Groups		Day 1		Day 8		Day 9		Day 10
		Electrodes implant		BUO surgery		Water or star fruit gavage		Water or star fruit gavage
WHI		-		-		-		-
WGV		-		-		Yes		Yes
SGV		-		-		Yes		Yes
NPW		-		Yes		Yes		Yes
NPS		Yes		Yes		Yes		Yes

The surgery was held on the 8th day on the NPW and NPS groups. The animals were anesthetized with dissociative general intramuscular anesthesia using ketamine hydrochloride (100 mg/mL) at a dose of 70 mg/kg body weight associated with xylazine hydrochloride (2 g/100 mL) at a dose of 10 mg/kg body weight. Then their abdominal cavity was clean and exposed to induced nephropathy by obstructing two ends of the ureter on both sides using a silk suture thread. A section in the ureter between the two suture points concluded the obstruction, therefore making the animals uremic. To finish the procedure, the incisions were sutured with nylon thread and topical iodopovidone antiseptic (Rioquímica) was applied to the skin, as described elsewhere by Achar *et al*.^13^.

### Gavages to administrate water or star fruit juice

The gavages were performed with the support of a metallic orogastric cannula and the administration of the content varied according to the groups (WGV and NPW received gavages with water and SGV and NPS received star fruit juice).

The water from the gavage (drinking water) was acquired from the same location as those that remained kept in the respective boxes of each animal. Same star fruit were purchased from local producers. The star fruit juice was produced by putting the fruit in the blender for a few moments, without adding water and the final product was passed through a 3-micron porosity filter paper. On the day after the bilateral ureter obstruction surgery, all animals received 1 mL of defined liquid through gavages, regardless of the experimental group and its weight. All the gavages started in the morning with 3-h breaks for up to 2 days, always checking and respecting the conditions presented by the animals. The administration schedules were at 7:30, 10:30, 13:30, 16:30 on the first day and at 7:30, 10:30, 13:30 on the second day. The animals who presented epileptic seizures were perfused 1 h and 30 min after the crisis beginning and those with no seizures were perfused 1 h and 30 min after the last gavage on the second day.

### Dosage of urea and creatinine (day 10)

The animals were anesthetized with general dissociative intramuscular anesthesia using ketamine hydrochloride (100 mg/mL) at a dose of 70 mg/kg body weight associated with xylazine hydrochloride (2 g / 100 mL) at a dose of 10 mg/kg body weight. The rib cage and heart were exposed and approximately 3 mL of blood were removed from the left ventricle in order to measure the animal’s urea and creatinine. After this procedure, a transcardiac perfusion was performed with buffered saline (pH 7.4) and 4% paraformaldehyde fixing solution (PFA) at 4 °C for future analysis of brain material. The procedure for dosing urea and creatinine in order to prove the efficiency of the BUO surgery and the animal’s nephropathy situation. For this purpose, the commercial kit Urea CE (Labtest Diagnóstica) was used for urea, through the enzymatic-colorimetric system in serum samples by end-point reaction. For creatinine, a commercial kit Creatinine K (Labtest Diagnóstica) was used; this kit uses the enzymatic-colorimetric system in serum samples by kinetic reaction. All procedures for dosing urea and creatinine were strictly followed as indicated by the manufacturer.

### Electroencephalogram

In the NPS group, the electroencephalographic records were taken after the electrodes’ implantation. The animals were anesthetized on day 1 with general dissociative intramuscular anesthesia using ketamine hydrochloride (100 mg/mL) at a dose of 70 mg/kg body weight associated with xylazine hydrochloride (2 g/100 mL) at a dose of 10 mg/kg body weight and placed on the stereotactic equipment (KOPF brand); they had their head skin sectioned with a sagittal cut and the bregma point was identified (considered the point 0.0 for both anteroposterior [AP], lateral [LAT] and depth [DEP]). The following structures were arranged to assess seizures: hippocampus CA1 (AP: –4.6, LAT: 2.5, DEP: 3.0) and primary motor cortex (AP: –2.0, LAT: 1.5, DEF: 2.0). After this procedure, the animals remained in their usual cages for 7 days to reduce a possible inflammatory process caused by the implantation surgery and consequently an error during the search for electroencephalographic signals. On the 10th day, the animals were placed on a Faraday cage (built using acrylic material and covered with a thin metallic mesh) and their records were made using the Nihon Kohden 4412P Neurofax device. An evaluator remained monitoring and observing the animals’ behavior during this period.

### Data analysis

The values obtained from dosages of urea and creatinine were analyzed statistically using ANOVA test, to identify if there were differences between the studied groups, being considered significant if p < 0.01. Differences between groups were individually analyzed using the Student’s t-test, being considered significant if p < 0.01.

## Results

### Dosage of urea and creatinine

**Figure 1:**
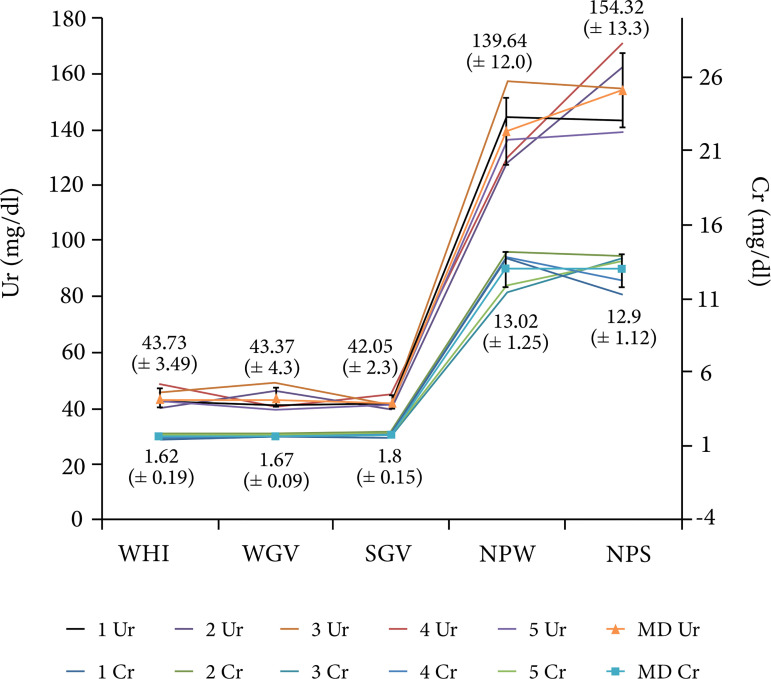
Dosage of urea and creatinine in each of the experimental animals. In the line representing the average dosages of urinary and creatinine in each of the experimental groups, the standard deviation was also expressed graphically in the form of a vertical bar, being the mean (± standard deviation) also expressed numerically above the lines referring to urea and below the lines referring to creatinine. Shaw group (WHI); water gavage group (WGV); star fruit gavage group (SGV); néphropathie group with water gavage (NPW); néphropathie group with star fruit gavage (NPS); urea (Ur); ereatinine (Cr).

Regarding the creatinine dosage (expressed as mean ± standard deviation), the WHI, WGV and SGV group did not reach about 2 mg/dL WHI = 1.62 ± 0.19, WGV = 1.67 ± 0.09 and SGV = 1.80 ± 0.15; however, in the NPW and NPS groups, the dosages approached 13 mg/dL (NPW = 13.02 ± 1.25 and NPS = 12.90 ± 1.12), which demonstrated a statistically greater value. Regarding the urea measurements (expressed as mean ± standard deviation), the WHI, WGV and SGV groups were around 43 mg/dL (WHI = 43.73 ± 3.49, WGV = 43.37 ± 4.30 and SGV = 42.05 ± 2.30), and the animals in the NPW and NPS group around 150 mg/dL (NPW = 139.64 ± 12.00 and NPS = 154.23 ± 13.30). Figure 1 shows the urea and creatinine dosage data for each of the experimental animals as well as the mean and standard deviation in each experimental group.

### Electroencephalogram and behavioral observation

The NPS group showed different behavioral manifestations from the other groups since the first gavage. Initially, there was only discreet piloerection and the abandonment of self-cleaning. There was a drop in exploratory activity and the animals tended to remain quiet. On the second day of gavages (day 10), progressively and mainly after the third gavage, in addition to piloerection and relative quieting, there is an important sialorrhea and masticatory automatism. In fact, this evolution occurred, but for a very intense alert, probably reflecting an important increase in nervous excitability related to star fruit. Over time, the animals changed from the previous state of stillness and piloerection to agitation, progressing to myoclonus and subsequently generalized tonic-clonic seizures became predominant.

**Figure 2:**
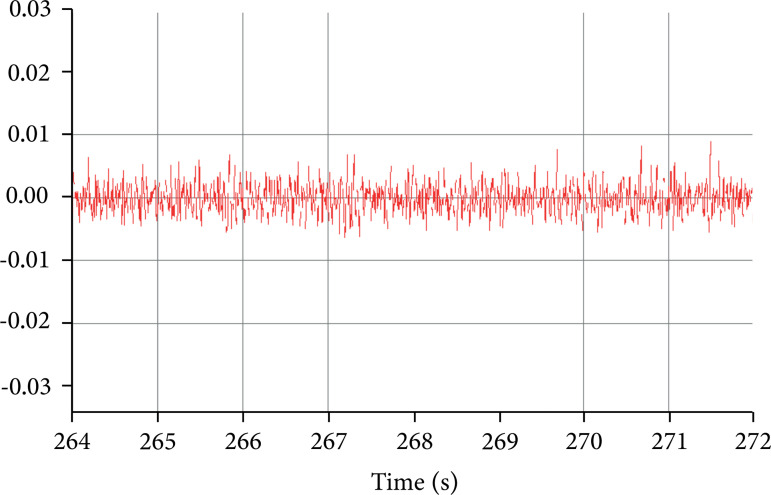
Electroencephalogram of the left cerebral cortex of an NPS group animal after the first gavage. Despite being present, theta waves have low regularity and rare clustering. Between 265 and 266 s, the presence of spindles shows a characteristic of relaxed wakefulness.

The visual analysis of the EEGs of the NPS group initially (10th day in the morning) showed the characteristic patterns of wakefulness, with greater evidence of theta waves and the presence of faster rhythms and desynchronization secondarily. No spicules or other potentials with pathological characteristics were observed in this condition, what was known as a relaxed wakefulness (Fig. 2).

Over time, wave characteristics have changed. This occurred in both cortical and hippocampal registers. It is noted that theta waves and desynchronization are present, but no clusters or visual evidence of modulation of these potentials were observed, making it possible to compare this pattern with relaxed wakefulness. This comparison reinforces the small spindles that eventually appeared. The animals, in this phase of the experiments, were prostrate and did not perform self-cleaning behavior.

Progressively, it is noticed in nephropathic animals that the electrical activity resembles even more that of waking, this time active, with theta waves grouped and with a high degree of regularity, with desynchronization almost absent. However, these outbreaks of theta waves differ from normal alert because they extend far beyond the characteristic few seconds, sometimes reaching more than a minute. In these circumstances, the animals presented, in addition to piloerection, sialorrhea and masticatory automatism, irregular muscular concussions, more evident in the limbs, which were classified as myoclonus. They were also more restless, appearing to explore the cage at random.

**Figure 3:**
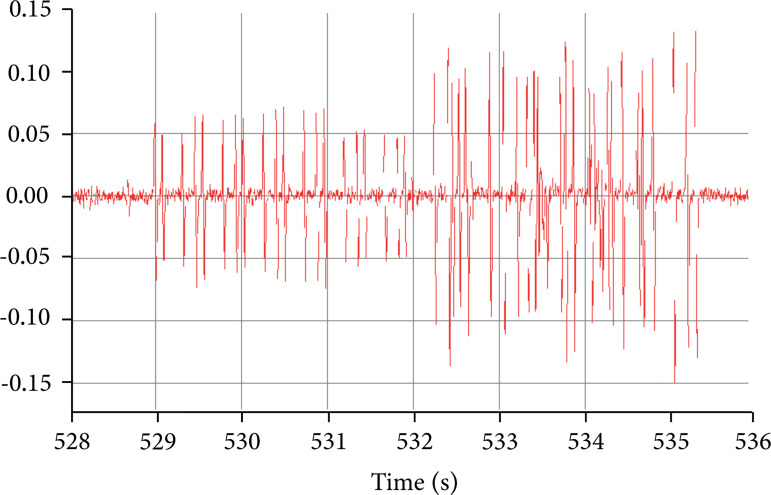
Electroencephalogram after the fourth gavage on the animal NPS. Prolonged spicules are observed with little organization. It is suggested that the entire nervous system is mobilized and it appears that it is not possible to define migrations, that is, whether they are cortical or between cortical and hippocampal.

Even after the third gavage of the second day, changes in the EEG, with an evident outbreak of high voltage spikes, not synchronous between the registered structures, were noticed. There is almost complete suppression of patterns suggestive of alertness and, in all channels, a spike of spikes. The components of these cluster did not show bilateral synchrony, but between the ipsilateral cortex and hippocampus. The electrical activity progressively lost its visible synchrony and spiculate potentials of great amplitude appear, in weakly regular volleys. This disorganization occurred in a generalized way, without it being visually possible to distinguish any migration between the cortical leads or between the cortical and the hippocampal leads, which suggests that the origin of the crises is multifocal. The animals remained more agitated and, suddenly, presented generalized tonic-clonic seizures, which were initially brief; however, after increasing in frequency and amplitude, they became continuous (Fig. 3).

## Discussion

Star fruit is a sweet and sour fruit, rich in minerals (calcium, potassium, sodium, phosphorus, magnesium, iron, copper, zinc and manganese) vitamins A, C, B complex and oxalic acid^1^^,^ 14.

In 1980, Muir and Lam^14^ first reported a possible toxicity related to some active component present in the star fruit, causing a depressive effect on the central nervous system of healthy mice. The first neurotoxic consequences from the administration of intraperitoneal injections of star fruit juice in nephropathic rats generated the induction of convulsive crises suggesting, then, the supposed existence of a neurotoxicity from the fruit^14^.

In 1993, Martin *et al.* 3 presented the first report where uremic patients developed the common clinical sign of incoercible hiccup right after ingesting star fruit juice. Since then, the number of cases and reports such as those of has increased^15^^–^ 19.

Poisoning by eating star fruit can be clinically expressed for hours or days and is classified into three levels that vary according to the clinical manifestations presented by patients. Mild intoxication is characterized by mild and intractable hiccups, vomiting and insomnia. In moderate intoxication, psychomotor agitation, confusion, paresthesia and numbness of the limbs are observed. In the most severe cases, that is, in severe intoxication, moderate to severe mental confusions with evolution to coma are observed, seizures evolving to epileptic status, hemodynamic instability progressing to hypotension and shock, even death (where 75% of mortality can occur in cases involving the epileptic state)^17^^–^ 21.

Some clinical studies found that the presence of signs and symptoms caused by the toxicity coming from the fruit are related to the level of intoxication triggered^4^^,^ 5 ^,^ 20 ^,^ 21. Therefore, the current study helps to corroborate this affirmative, since an increase in the pathological signs in animals and the onset of convulsive crises resulting from neurointoxication were noticed during each gavage.

In the clinical cases reported by Moyses Neto^17^, it was apparent that there was no direct relationship between the amount of star fruit ingestion and the severity of intoxication. Through this, it was considered an animal weight without great variations and 1 mL of constant star fruit juice for all gavages, for all periods.

Considering all this relationship, when ingesting star fruit juice, patients considered normal (in terms of kidney functions) are able to easily absorb, distribute and excrete the toxin through the renal pathways, without any change in their body. On the other hand, it was considered if the data presented, nephropathic patients have difficulties during the absorption and, mainly, excretion processes, because the toxin, instead of being removed from the body, continues to cause an increase in serum levels. As a result, patients may experience severe signs of intoxication, such as epileptic seizures.

Studies have shown that the neurotoxin of star fruit would cause an inhibition between the binding of GABA in its receptors. The same did not occur in the ionotropic glutamate type receptors, however, for both cases, the release processes and dependent sodium uptake showed no significant changes. Thus, it is believed that the onset of convulsive crises immediately after eating the star fruit may be linked to the blockage of GABAergic receptors, causing an imbalance between the neurotransmitter systems and increased cerebral excitability^8^^–^ 10.

The pathogenesis of the seizure process is understood as an imbalance of the excitatory neurotransmission system, which generates local or generalized hyperexcitability in the central nervous system. Evidence points out that the triggers of seizures would be involved with the changes caused in the neurotransmission systems^8^.

The neurological destabilization triggered by the neurotoxin generates an increase in brain excitability that sets off seizures, which can happen in an unpredictable way and in most cases are even fatal. Taking into consideration the given information, the EEG has become extremely important and useful for epileptic assessment. With the results obtained from records, dysrhythmias associated with epilepsy were identified according to the amount of star fruit juice ingested. After the first and second gavage, low regularity theta waves were observed, as well as asynchronization between them and the absence of spindles, which indicated that the animal was in a relaxed state. From the third gavage on, characteristic patterns of abnormal theta rhythm (more grouped theta waves), increased amplitude, greater number of spicules and common epilepsy behaviors (sialorrhea, reduced activity, myoclonus, agitation, tonic-clonic crises) were detected, due to neuronal activation and agitation that arise from intoxication.

Considering that seizures are not present in healthy individuals who consume star fruit or in nephropathic patients, the use of EEG only in the NPS group was intended to validate the behavioral observation of this group’s convulsive condition, which presents the association between nephropathy and star fruit, an event clinically reported only in this type of association. Thus, it was not necessary to analyze the EEG in the other groups (WHI, WGV, SGV and NPW) as they are not clinically associated^15^^–^ 19.

Urea and creatinine are used as biomarkers for the evaluation of renal function, so, in order to validate the experimental model, both dosages were performed by blood analysis. As described in the literature^3^^,^ 11 ^,^ 16, it was already expected that the biomarker levels would be above the reference values. With that, in the case of animals who underwent BUO surgery (NPW and NPS), the values were increased, thus confirming the nephropathic condition. While the gavages were ministered, the animals started to present progressive characteristic patterns of epilepsy, both in physical form (signs and symptoms) and in the EEG results, thus demonstrating the presence of epileptic crises associated with star fruit intoxication.

This study’s data show that the BUO surgery reproduced a qualified experimental model for the study, in addition to elucidating that the gavages with star fruit juice were the triggering aspect regarding the seizures.

## Conclusion

The standardization of the experimental model was efficient in demonstrating a correlation between neurotoxicity, star fruit and nephropathy, therefore serving as an important model to further researches.
